# Temporary sphincter-preserving covered biliary stent with a frontal umbrella-shaped occlusive mechanism for common bile duct stone removal

**DOI:** 10.1055/a-2813-3282

**Published:** 2026-03-31

**Authors:** Wengang Zhang, Hongyi Sun, Qingzhen Wu, Haoqi Zhai, Bozong Shao, Enqiang Linghu

**Affiliations:** 1 Senior Department of Gastroenterology, The First Medical Center of Chinese PLA General Hospital, Beijing, China

**Keywords:** Pancreatobiliary (ERCP/PTCD), Stones, ERC topics, Cholangioscopy

## Abstract

Currently, endoscopic retrograde cholangiopancreatography (ERCP) has been established as standard treatment for common bile duct (CBD) stones. However, this procedure requires endoscopic sphincterotomy (EST), which compromises function of the sphincter of Oddi and may lead to adverse events (AEs) such as bleeding, perforation, biliary reflux of intestinal contents, and stone recurrence. It is particularly unsuitable for patients requiring long-term anticoagulation therapy. In light of this, several researchers, including our team, have attempted to place a self-expandable metal stent (SEMS) in the distal CBD prior to stones clearance as an alternative to EST. Nevertheless, widespread adoption of this strategy has been limited by the issue of stone impaction between the stent and CBD wall. To address this, our team developed a temporary sphincter-preserving covered biliary stent (TSP-CBS) device equipped with a frontal umbrella-shaped occlusive mechanism. The open/close operation of this umbrella component effectively prevents stone impaction as described above. This study first validated safety of TSP-CBS in four porcine models, with no intraoperative or postoperative AEs observed. Subsequently, we performed two clinical cases of EST-free ERCP using TSP-CBS for CBD stone removal. Both procedures successfully cleared the stones. One patient developed postoperative hyperamylasemia, which resolved with conservative management, and no other intraoperative or postoperative AEs occurred. In summary, this preliminary study demonstrates the safety and feasibility of TSP-CBS for performing EST-free ERCP in patients with CBD stones. Further validation in larger clinical cohorts is warranted.

## Introduction

**Fig. 1:**
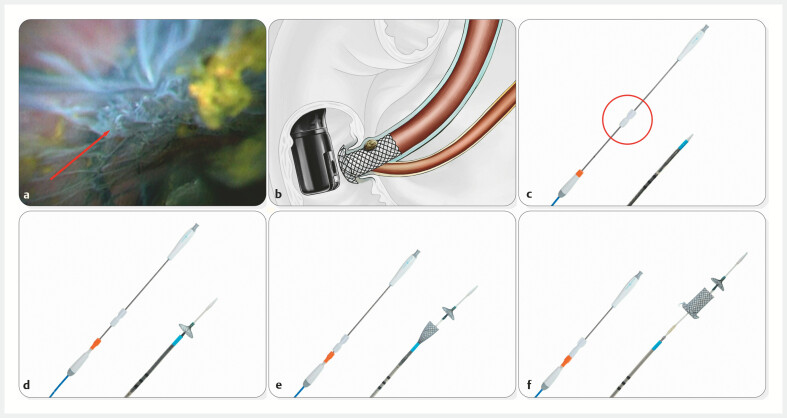
Representative images of stone impaction between the stent and the common bile duct (CBD) wall and the temporary sphincter-preserving covered biliary stent (TSP-CBS). **a** Stone impaction between the stent and the CBD wall. **b** Schematic diagram of stone impaction between the stent and the CBD wall. **c** Overall structure of the TSP-CBS, including a close-up view highlighting the safety lock mechanism on the handle. **d** Deployment of the frontal umbrella-shaped occlusive device of the TSP-CBS. **e** Overall structure of the TSP-CBS, including a close-up view showing the stent partially deployed. **f** Overall structure of the TSP-CBS, including a close-up view highlighting the stent in its fully deployed configuration.

**Fig. 2:**
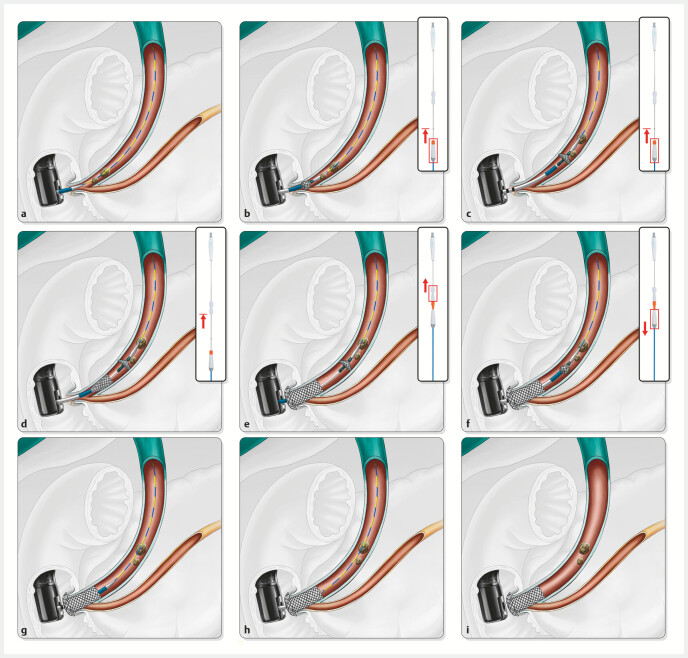
Schematic illustrating the deployment process of the temporary sphincter-preserving covered biliary stent (TSP-CBS) in a common bile duct (CBD) with stones. **a** Advancing the TSP-CBS over a guidewire toward the distal portion of the CBD. **b** Retracting the anterior handle toward the black marker point to gradually deploy the frontal umbrella-shaped occlusive device distal to the stone. **c** With the frontal umbrella-shaped occlusive device deployed, the TSP-CBS is further advanced over the guidewire to the target deployment site, while the stone is simultaneously pushed toward the proximal CBD. **d** The anterior handle is moved from the black marker to the safety lock position to gradually deploy the proximal segment of the stent, while the stone, positioned above the umbrella-shaped occlusive device, is prevented from becoming impacted between the stent and the duct wall. **e** After releasing the safety lock, retracting the anterior handle toward the posterior handle completes stent deployment. **f,g** The anterior handle is moved away from the posterior handle to retract the frontal umbrella-shaped occlusive device. **h,i** The outer sheath and guidewire are withdrawn together following the retrieval of the frontal umbrella-shaped occlusive device, thereby completing the TSP-CBS deployment procedure.

Endoscopic retrograde cholangiopancreatography (ERCP) is standard treatment for common bile duct (CBD) stones but requires endoscopic sphincterotomy (EST) 1 2 3 , resulting in sphincter dysfunction and some complications 4 . Furthermore, EST is contraindicated with anticoagulants. Consequently, multiple groups, including ours, have investigated placement of a self-expandable metal stent (SEMS) prior to stones clearance to avoid EST 5 6 . Nonetheless, stones often impact between the stent and CBD wall ( Fig. 1 **a,** Fig. 1 **b** ). To address this, our team developed a temporary sphincter-preserving covered biliary stent (TSP-CBS) device equipped with a frontal umbrella-shaped occlusive mechanism ( Fig. 1 **c,** Fig. 1 **d,** Fig. 1 **e,** Fig. 1 **f** ). The open/close operation of this umbrella component effectively prevents stone impaction as described above ( Fig. 2 ). Because TSP-CBS had not been previously applied in clinical practice, we first conducted an animal study using Panama pigs to preliminarily assess whether the device could be successfully deployed during ERCP—particularly under flexed duodenoscope conditions—and whether it might cause bile duct injury. The results were satisfactory. Subsequently, we carried out a clinical study to evaluate the safety and feasibility of TSP-CBS for performing EST-free ERCP in patients with CBD stones.

This study was approved by the Ethics Committee of the Chinese PLA General Hospital. Informed consent was obtained from all participating patients.

## Methods

### Animal model

**Fig. 3:**
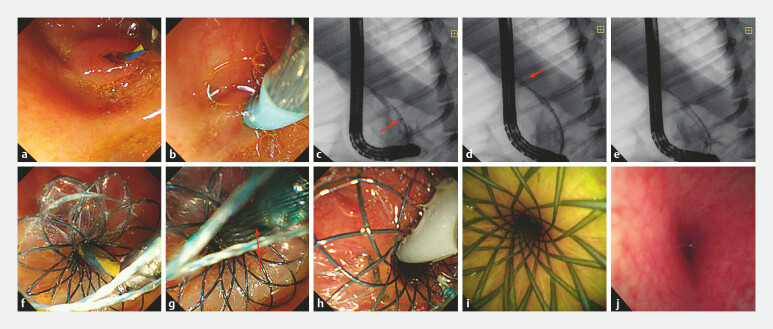
The deployment process of the temporary sphincter-preserving covered biliary stent (TSP-CBS) in a porcine model. **a** Biliary cannulation was conducted. **b** Advancing the TSP-CBS over a guidewire toward the distal portion of the common bile duct (CBD). **c,d** With the frontal umbrella-shaped occlusive device deployed (red arrows), the TSP-CBS is further advanced over the guidewire to the target deployment site. **e,f** The stent is fully deployed. **g** The umbrella-shaped occlusive device was collapsed and retrieved (red arrows). **h-j** Subsequent single-operator cholangioscopy (SOC) confirmed the absence of biliary mucosal injury.

Prior to clinical application, we validated safety and feasibility of TSP-CBS deployment in the CBD following the procedure illustrated in Fig. 2 using four Panama pigs ( Fig. 3 ). Key steps in deployment of TSP-CBS are shown in Table 1 . Post-deployment single-operator cholangioscopy (SOC) was performed to evaluate integrity of the CBD mucosa ( Fig. 3 **h,** Fig. 3 **i,** Fig. 3 **j** ).

After the procedure, the pigs were fasted for 24 hours, followed by a liquid diet for another 24 hours. A normal diet was gradually reintroduced starting on the third day.

**Table:** **Table 1** Key steps in deployment of a temporary sphincter-preserving covered biliary stent (TSP-CBS).

**Procedure steps**	**Operation**	**Schematic diagram**
Step 1	Advancing the TSP-CBS over a guidewire toward the distal portion of the common bile duct (CBD).	Fig. 2 **a**
Step 2	Retracting the anterior handle toward the black marker point to gradually deploy the frontal umbrella-shaped occlusive device distal to the stone.	Fig. 2 **b**
Step 3	With the frontal umbrella-shaped occlusive device deployed, the TSP-CBS is further advanced over the guidewire to the target deployment site, while the stone is simultaneously pushed toward the proximal CBD.	Fig. 2 **c**
Step 4	The anterior handle is moved from the black marker to the safety lock position to gradually deploy the proximal segment of the stent, while the stone, positioned above the umbrella-shaped occlusive device, is prevented from becoming impacted between the stent and the duct wall.	Fig. 2 **d**
Step 5	After releasing the safety lock, retracting the anterior handle toward the posterior handle completes stent deployment.	Fig. 2 **e**
Step 6	The anterior handle is moved away from the posterior handle to retract the frontal umbrella-shaped occlusive device.	Fig. 2 **f,** Fig. 2 **g**
Step 7	The outer sheath and guidewire are withdrawn together following retrieval of the frontal umbrella-shaped occlusive device, thereby completing the TSP-CBS deployment procedure.	Fig. 2 **h,** Fig. 2 **i**

### Clinical study

Patients with CBD stones who underwent evaluation for this study were enrolled based on the following criteria: Only patients who met all of the following criteria were included: 1) age between 18 and 80 years; 2) considered at high risk for post-EST bleeding due to long-term anticoagulant use, thrombocytopenia, or the presence of a periampullary diverticulum; and 3) stone diameter ≥ 1.0 cm. Patients were excluded if they met any of the following conditions: 1) age < 18 years or > 80 years; 2) severe underlying comorbidities precluding tolerance to ERCP; or 3) refusal to provide written informed consent.

#### Case 1

**Fig. 4:**
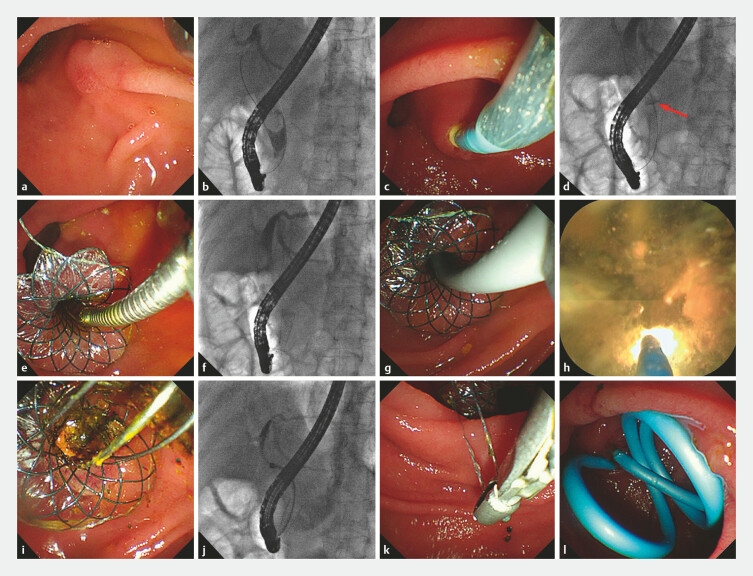
The endoscopic sphincterotomy (EST)-free ERCP procedure with adjunctive single-operator cholangioscopy (SOC) for common bile duct (CBD) stones, utilizing the temporary sphincter-preserving covered biliary stent (TSP-CBS) (Case 1). **a,b** Biliary cannulation was conducted, followed by cholangiography which revealed a solitary 2.0-cm stone in the distal CBD. **c** The TSP-CBS was deployed over a guidewire into the CBD, followed by expansion of its frontal umbrella-shaped occlusive device below the stone. **d** During advancement to the deployment site, the expanded umbrella-shaped occlusive device (red arrows) of the TSP-CBS proximally displaces the stone, thus enabling safe stent deployment without risk of stone entrapment between the stent and the CBD wall. **e,f** The umbrella-shaped occlusive device was collapsed and retrieved. **g,h** SOC-guided electrohydraulic lithotripsy (EHL) was performed through the TSP-CBS. **i** Fluoroscopy-guided basket extraction. **j** SOC confirmation of stone-free biliary duct. **k** The TSP-CBS was removed using grasping forceps. **l** Deployment of a self-release biliary plastic stent.

A female patient with a CBD stone was admitted to our institution. Preoperative evaluation revealed a stone approximately 2.0 cm in size and a periampullary diverticulum. Given that EST would require a large incision with associated risks of postoperative perforation or bleeding, an EST-free ERCP through the TSP-CBS, with adjunctive SOC for stone extraction, was performed. The procedure was performed as follows. First, biliary cannulation was conducted, followed by cholangiography which revealed a solitary 2.0-cm stone in the distal CBD ( Fig. 4 **a,** Fig. 4 **b** ). Second, the TSP-CBS was deployed over a guidewire into the CBD ( Fig. 4 **c** ), followed by expansion of its frontal umbrella-shaped occlusive device below the stone. Third, the TSP-CBS was advanced to its predetermined deployment site, during which the expanded umbrella-shaped occlusive device (red arrows) of the TSP-CBS proximally displaces the stone, thus enabling safe stent deployment without risk of stone entrapment between the stent and the CBD wall ( Fig. 4 **d** ). Fourth, the umbrella-shaped occlusive device was collapsed and retrieved ( Fig. 4 **e,** Fig. 4 **f** ). Fifth, SOC-guided electrohydraulic lithotripsy (EHL) was performed through the TSP-CBS ( Fig. 4 **g,** Fig. 4 **h** ). Sixth, fluoroscopy-guided basket/balloon extraction was conducted ( Fig. 4 **i** ), with subsequent SOC confirmation of stone-free biliary duct ( Fig. 4 **j** ). Finally, the TSP-CBS was removed using grasping forceps ( Fig. 4 **k** ), followed by deployment of a self-release biliary plastic stent ( Fig. 4 **l** ).

#### Case 2

The endoscopic sphincterotomy (EST)-free ERCP procedure for common bile duct (CBD) stones, utilizing the temporary sphincter-preserving covered biliary stent (TSP-CBS) (Case 2).Video 1

**Fig. 5:**
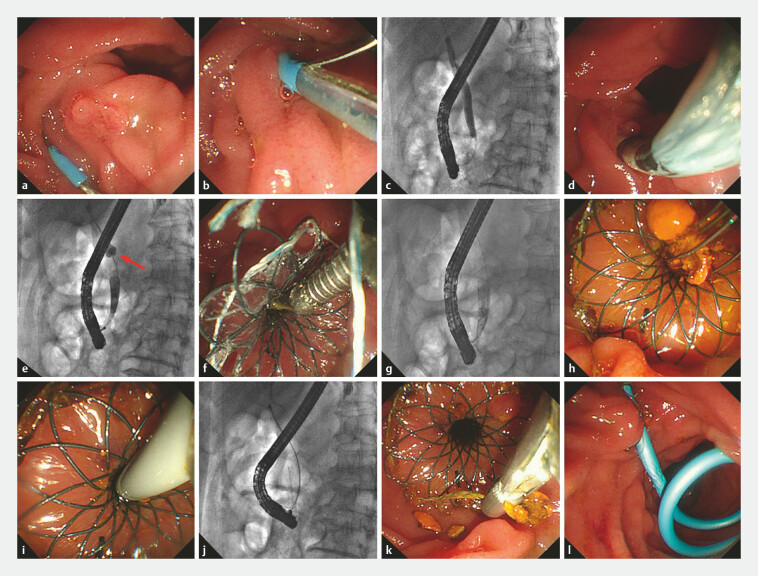
The endoscopic sphincterotomy (EST)-free ERCP procedure for common bile duct (CBD) stones, utilizing the temporary sphincter-preserving covered biliary stent (TSP-CBS) (Case 2). **a,b,c** Biliary cannulation was conducted, followed by cholangiography which revealed a solitary 1.0-cm stone in the CBD. **d** The TSP-CBS was deployed over a guidewire into the CBD, followed by expansion of its frontal umbrella-shaped occlusive device below the stone. **e,f** During advancement to the deployment site, the expanded umbrella-shaped occlusive device (red arrows) of the TSP-CBS proximally displaces the stone, thus enabling safe stent deployment without risk of stone entrapment between the stent and the CBD wall. **g** The TSP-CBS was deployed completely after retrieval of the umbrella-shaped occlusive device. **h** Fluoroscopy-guided basket extraction. **i,j** SOC confirmation of stone-free biliary duct. **k** The TSP-CBS was removed using grasping forceps. **l** Deployment of a self-release biliary plastic stent.

A 71-year-old male with a CBD stone, who was at elevated bleeding risk due to long-term oral anticoagulation for coronary heart disease, underwent an EST-free ERCP procedure for stone extraction at our institution. The procedure was performed as follows ( Fig. 5 , Video 1 ). First, biliary cannulation was conducted, followed by cholangiography, which revealed a solitary 1.0-cm stone in the CBD ( Fig. 5 **a,** Fig. 5 **b,** Fig. 5 **c** ). Second, the TSP-CBS was deployed, adhering to the same procedural protocol as detailed for Case 1 ( Fig. 5 **d,** Fig. 5 **e,** Fig. 5 **f,** Fig. 5 **g** ). Third, fluoroscopically-guided basket stone extraction was performed ( Fig. 5 **h** ), followed by SOC confirming complete ductal clearance ( Fig. 5 **i,** Fig. 5 **j** ). The TSP-CBS was then endoscopically retrieved using grasping forceps ( Fig. 5 **k** ), and a self-release biliary plastic stent was deployed ( Fig. 5 **l** ).

#### Postoperative management

Postoperatively, at 24 hours, patients underwent complete blood count and biochemical tests, with close monitoring of abdominal signs. If significant abdominal pain occurred, an abdominal CT scan was promptly performed. After the procedure, patients were managed with fasting, acid suppression therapy, and antibiotics as symptomatic treatment. If postoperative pancreatitis (PEP) occurred, somatostatin therapy was administered. In the absence of postoperative adverse events (AEs), oral intake was initiated with water on the third postoperative day, followed by a liquid diet on the fourth day, with gradual advancement to a normal diet.

Patients were scheduled for outpatient follow-up at 1 month postoperatively. If abdominal pain, fever, jaundice, or other abnormal signs occurred, an abdominal CT scan was performed.

### Endpoints and related definition

#### Primary and secondary endpoints

For the animal model study, the primary endpoints were the technical success rate of TSP-CBS deployment and the incidence of bile duct injury. For the clinical study, the primary endpoints were the technical success rate of TSP-CBS deployment, the incidence of bile duct injury, and the complete stone retrieval rate. The secondary endpoint was incidence of PEP.

### Definitions

Technical success of TSP-CBS deployment was defined as successful passage and deployment of the device following the described procedural steps, resulting in adequate opening of the CBD orifice at the major duodenal papilla. Bile duct injury was defined as presence of abnormalities such as mucosal abrasion or bleeding detected by SOC following TSP-CBS deployment. Complete stone retrieval was defined as clearance of all CBD stones using the access established by the TSP-CBS. PEP was defined according to the established consensus criteria 7 .

## Results

The main outcomes of the animal model and clinical studies are shown in Table 2 . Among a total of six procedures, including four animal experiments and two clinical cases, the technical success rate for TSP-CBS deployment was 100%, with a bile duct injury rate of 0%.

**Table:** **Table 2** Main outcomes of the animal model and clinical studies.

Outcomes of the animal model study, n = 4	
Technical success rate of TSP-CBS deployment, n (%)	4 (100%)
Bile duct injury rate, n (%)	0 (0%)
Postoperative adverse events, n (%)	0 (0%)
Outcomes of the clinical study, n = 2	
Technical success rate of TSP-CBS deployment, n (%)	2 (100%)
The complete stone retrieval rate, n (%)	2 (100%)
Bile duct injury rate, n (%)	0 (0%)
Postoperative hyperamylasemia, n (%)	1 (50%)

In the animal study, the TSP-CBS was successfully deployed in all four procedures. Subsequent SOC confirmed absence of bile duct injury ( Fig. 3 **h,** Fig. 3 **i,** Fig. 3 **j** ). All four animals recovered uneventfully. No procedure- or device-related AEs occurred intraoperatively or postoperatively.

In the clinical study, complete stone retrieval was achieved in both procedures. Patient 1 (Case 1) had an uneventful recovery, with no AEs identified by abdominal signs or imaging during 1-month follow-up. Patient 2 (Case 2) developed postoperative hyperamylasemia, which resolved with conservative management. No other AEs were identified during the subsequent 1-month follow-up based on abdominal signs or imaging.

## Discussion

Dynamic simulation demonstrating the deployment of a temporary sphincter-preserving covered biliary stent (TSP-CBS) in a stone-bearing common bile duct (CBD).Video 2

For years, multiple research groups, including ours, have attempted to utilize SEMS to enable EST-free ERCP for management of CBD stones. However, risk of stone impaction between the stent and the CBD wall ( Fig. 1 **a,** Fig. 1 **b** ) has hindered widespread clinical adoption of this strategy. This study introduces a novel TSP-CBS featuring an umbrella-shaped occlusive device ( Fig. 1 **d,** Fig. 1 **e,** Fig. 1 **f** ). By deploying this umbrella-shaped occlusive device distal to the stones prior to stent placement and subsequently closing and retrieving it after stent release ( Fig. 2 Video 2 ), the system effectively prevents the aforementioned issue of stone impaction ( Fig. 1 **a,** Fig. 1 **b** ).

Based on both animal experiments and clinical practice, this study provides initial evidence supporting feasibility and safety of TSP-CBS for performing EST-free ERCP in treatment of CBD stones. A major safety concern was potential risk of bile duct injury caused by the distal umbrella-shaped occlusive device during its advancement toward the hepatic hilum. The animal experiments demonstrated that the TSP-CBS could be deployed smoothly without complications. In this study, clinical practice further demonstrated that the TSP-CBS effectively prevents stone impaction between the stent and the CBD wall through controlled deployment and retrieval of its distal umbrella-shaped occlusion device.

The key advantage of EST-free ERCP lies in its potential to avoid EST-related AEs, such as bleeding, perforation, and stone recurrence. Therefore, we consider that EST-free ERCP via TSP-CBS is currently applicable to the following patients and may benefit them: 1) patients at high risk of post-EST bleeding due to long-term oral anticoagulant use or thrombocytopenia; and 2) patients at high risk of post-EST perforation or bleeding due to duodenal major papilla located adjacent to a diverticulum. In addition, TSP-CBS holds promise for addressing the technical challenges of ERCP stone extraction in patients with CBD stones accompanied by distal bile duct strictures. Regarding characteristics of CBD stones, given that smaller muddy stones may not be completely occluded by the umbrella-shaped device of TSP-CBS, we currently recommend applying this technique to CBD stones ≥ 1.0 cm.

Another technical advantage of EST-free ERCP via TSP-CBS is preservation of the sphincter of Oddi function postoperatively. Currently, endoscopic papillary balloon dilation (EPBD) or minimal EST combined with EPBD is also frequently employed during ERCP procedures to preserve papillary sphincter function 8 9 . Compared with EPBD or minimal EST + EPBD, TSP-CBS offers the following advantages. First, it facilitates straightening of the distal and intramural bile duct segments, thereby improving accessibility and maneuverability for stone extraction devices and the cholangioscope. Second, in cases requiring SOC-guided lithotripsy, the deployed TSP-CBS maintains a patent lumen between the cholangioscope and the bile duct wall. This design promotes adequate outflow of irrigated saline, effectively mitigating elevated intra-biliary pressure—a well-documented risk factor for post-procedure cholangitis as supported by previous literature 10 . Third, temporary dilation of the biliary sphincter induced by the TSP-CBS simplifies both stone extraction and removal of fragmented calculi. Furthermore, residual fragments can be effectively evacuated using endoscopic negative-pressure suction. Fourth, the positioned TSP-CBS helps avoid unintended cannulation of the pancreatic duct during subsequent therapeutic manipulations. Lastly, because minimal EST + EPBD inherently involves EST manipulation, it carries a certain risk of post-procedure papillary bleeding, whereas TSP-CBS can largely circumvent this complication.

Regarding stone extraction techniques for larger and more challenging CBD stones—as illustrated in Case 1—a combined approach involving SOC-guided lithotripsy followed by basket or balloon extraction under fluoroscopic guidance can be utilized following TSP-CBS deployment. For smaller stones, illustrated in Case 2, conventional extraction using baskets or balloons under fluoroscopic guidance is feasible following TSP-CBS deployment.

This study has a major limitation in its small clinical sample size, which included only two patients. However, to the best of our knowledge, this is the first report about clinical application of TSP-CBS. In addition to the aforementioned advantages, TSP-CBS has also incorporated the following ingenious design details in specific aspects: First, a dome-shaped flange at the duodenal end of the stent ( Fig. 4 **e** ) minimizes risk of proximal migration toward the CBD caused by frictional forces during repeated instrument passage. Second, the two-stage deployment mechanism, equipped with a safety lock on the handle ( Fig. 1 **c** ), provides controlled stent release and prevents accidental dislodgement into the CBD. Another important point that must be addressed is that, given the limited number of cases included in this study, potential AEs such as bile leakage and biliary mucosal injury associated with use of TSP-CBS may not have been fully revealed. Therefore, at this stage, it is recommended that the procedure be performed by endoscopists with extensive experience in ERCP, specifically those who have performed over 1,000 procedures.

The primary aim of this study was to preliminarily demonstrate feasibility and safety of performing EST-free ERCP using TSP-CBS for CBD stones. Currently, a prospective clinical trial has been registered to conduct a study with a larger sample size (n = 40) to more comprehensively validate safety, feasibility, and efficacy of this technique.

## Conclusions

In summary, this study preliminarily demonstrates safety and feasibility of TSP-CBS-assisted EST-free ERCP for management of CBD stones.
